# Rapid valve sterilization with meropenem plus ceftolozane/tazobactam combination therapy for *Pseudomonas aeruginosa* prosthetic valve endocarditis

**DOI:** 10.1093/jacamr/dlaf112

**Published:** 2025-06-26

**Authors:** Valliammai Alaguvel, Anuj K Khetarpal, Allen Jankeel, Wendy A Tapia-Cano, Gabriela Martinez, Arianna Lorenzana, Zoe Hsiao, Warren Rose, George Sakoulas, Erlinda R Ulloa

**Affiliations:** Department of Pediatrics, University of California Irvine School of Medicine, Irvine, CA 92697, USA; Department of Pediatrics, University of California Irvine School of Medicine, Irvine, CA 92697, USA; Department of Pediatrics, University of California Irvine School of Medicine, Irvine, CA 92697, USA; Department of Pediatrics, University of California Irvine School of Medicine, Irvine, CA 92697, USA; Department of Pediatrics, University of California Irvine School of Medicine, Irvine, CA 92697, USA; Department of Pediatrics, University of California Irvine School of Medicine, Irvine, CA 92697, USA; Department of Pediatrics, University of California Irvine School of Medicine, Irvine, CA 92697, USA; School of Pharmacy, University of Wisconsin-Madison, Madison, WI 53705, USA; Collaborative to Halt Antibiotic-Resistant Microbes (CHARM), Department of Pediatrics, University of California San Diego School of Medicine, La Jolla, CA 92093, USA; Division of Infectious Diseases, Sharp Rees-Stealy Medical Group, San Diego, CA 92123, USA; Department of Pediatrics, University of California Irvine School of Medicine, Irvine, CA 92697, USA; Division of Infectious Diseases, Children’s Hospital of Orange County, Orange, CA 92868, USA

## Abstract

**Background:**

*Pseudomonas aeruginosa* infective endocarditis (IE) presents a significant clinical challenge, leading to high rates of treatment failure and mortality. Even with the use of antipseudomonal β-lactams combined with aminoglycosides or fluoroquinolones, these therapies often fail to provide clinical resolution and are frequently accompanied by severe adverse effects.

**Methods:**

We report a case of *P. aeruginosa* prosthetic valve endocarditis successfully treated with a combination of meropenem and ceftolozane/tazobactam. To investigate the synergistic effects of this combination, we conducted checkerboard, time-kill, human whole blood killing, and biofilm assays, as well as a simulated endocardial vegetation (SEV) model.

**Results:**

Meropenem plus ceftolozane/tazobactam combination therapy successfully bridged the patient to cardiac surgery, achieving rapid microbiological clearance and sterile intraoperative valve cultures. While checkerboard assays showed additivity, time-kill assays with subtherapeutic antibiotic concentrations did not demonstrate synergy in standard media. However, significant synergy was observed in human whole blood and biofilm environments, with modestly improved activity in the SEV model.

**Conclusions:**

The combination of meropenem and ceftolozane/tazobactam demonstrates promising synergy in physiologically relevant conditions, offering a potentially safer alternative for treating *P. aeruginosa* IE and stabilizing complex patients prior to cardiac surgery. Further clinical investigation is needed to evaluate its efficacy and safety profile in severe *Pseudomonas* infections, including IE.

## Introduction

*Pseudomonas aeruginosa* infective endocarditis (IE) is a rare but challenging infection, with high mortality rates, especially in cases of prosthetic valve endocarditis (PVE).^1^ Biofilm formation further complicates treatment by shielding bacteria from antibiotics and host immune responses, making eradication efforts significantly more difficult. Standard management includes cardiac surgery alongside combination therapy with antipseudomonal β-lactams and aminoglycosides or fluoroquinolones.^2^ However, aminoglycosides are limited by nephrotoxicity and ototoxicity, while fluoroquinolones are associated with tendon damage, neurotoxicity, and QTc prolongation.^3^ These challenges are amplified when surgery is not feasible due to clinical instability, highlighting the need for effective medical management strategies with improved safety profiles.

In this report, we present a case of *P. aeruginosa* PVE cured with meropenem plus ceftolozane/tazobactam. To understand the mechanisms behind this success, we conducted *in vitro* studies, including checkerboard, time-kill, human whole blood killing, and biofilm assays, along with a simulated endocardial vegetation (SEV) model to mimic *in vivo* therapeutic exposures.

## Materials and methods

### Informed consent and institutional approval

Informed consent for blood collection was obtained from healthy subjects under an approved UCI IRB protocol.

### Bacterial strains and antibiotics

Experiments were conducted using the *P. aeruginosa* bloodstream isolate PsA-DA, obtained from a patient with PVE as described below, and *P. aeruginosa* strains AR-0258 and AR-0259 from the CDC and FDA Antibiotic Resistance Isolate Bank. MIC data are in Table S1 (available as Supplementary data at *JAC-AMR* Online). Bacteria were grown overnight in LB and stored in 40% glycerol at −80°C. Fresh colonies were streaked weekly onto LA plates. Antibiotics stocks were prepared in molecular-grade water and stored at −20°C.

### *In vitro* studies and time-kill assays

MICs were determined by broth microdilution in cation-adjusted Mueller–Hinton broth (CA-MHB; Difco), following CLSI guidelines.^4^ Antibiotic activity was evaluated under standard (10^5^ cfu/mL) and high (10^7^ cfu/mL) inocula to simulate biofilm-related infection burdens. MBCs were defined as the lowest concentration killing ≥99.9% of the inoculum.^5^ Checkerboard assays assessed antibiotic interactions, defined by fractional inhibitory concentration indices (FICIs): synergy (≤0.50), additivity (>0.50–1.0), indifference (>1–4), and antagonism (>4).^5^ Time-kill assays were performed only under high inoculum in CA-MHB and human whole blood with subtherapeutic antibiotic concentrations.^6^ Bacterial viability was assessed at 6 and 24 h. Synergy was defined as a ≥2 log_10_ cfu/mL reduction versus the most active agent and a ≥1 log_10_ cfu/mL reduction from baseline.^5^

### Biofilm assays

Biofilm assays were performed as previously described.^6^ Overnight LB cultures were diluted 1:200 in CA-MHB to OD_600_ = 0.40. Ninety-six-well collagen-coated plates were inoculated with 100 μL of bacterial suspension (2 × 10^8^ cfu/mL) and incubated at 37°C for 48 h. Plate absorbance at OD₆₀₀ was measured before antibiotic exposure to confirm equivalent biofilm formation. After removing planktonic bacteria and washing with PBS, CA-MHB with or without antibiotics (meropenem, ceftolozane/tazobactam, or both) was added. Plates were incubated for another 48 h; then, adherent cells were fixed, stained with 0.1% safranin, and quantified by OD_490_ after acetic acid extraction. Alternatively, biofilms were disrupted, serially diluted, and plated to enumerate viable bacteria. Biofilm eradication was defined as the lowest test concentration yielding no growth. For biofilm inhibition assays, antibiotics were added with bacteria during the initial 48 h incubation, followed by washing and cfu enumeration or staining.

### Pharmacodynamic SEV model

A two-compartment *ex vivo* SEV model simulated human-like antibiotic exposures.^7^ SEVs were prepared with pooled human cryoprecipitate, platelets, bovine thrombin, and aprotinin, yielding 3–3.5 g/dL albumin and 6.8–7.4 g/dL total protein, reflecting human physiological levels.^8^ SEVs were added 30 min before antibiotic dosing, with continuous media infusion simulating human pharmacokinetics. Experiments were performed in duplicate, with two SEVs collected per time point (*n* = 4). SEVs were digested with trypsin (25 mg/mL), vortexed for 1 h, plated, and expressed as log_10_ cfu/g of tissue. Human therapeutic regimens (meropenem 2 g q8h and ceftolozane/tazobactam 2 g/1 g q8h) were simulated using published pharmacokinetic data from FDA-approved labelling, with 1 h infusions under normal renal function (creatinine clearance >50 mL/min), and were adjusted for unbound drug levels accounting for SEV protein binding.^9,10^

### Statistical analyses

GraphPad Prism version 10.4.2 was used. Two-group comparisons used unpaired two-tailed *t*-tests; comparisons involving more than two groups used one- or two-way ANOVA with *post hoc* Tukey’s test. *P* < 0.05 was considered significant.

## Results and discussion

**Figure 1.**
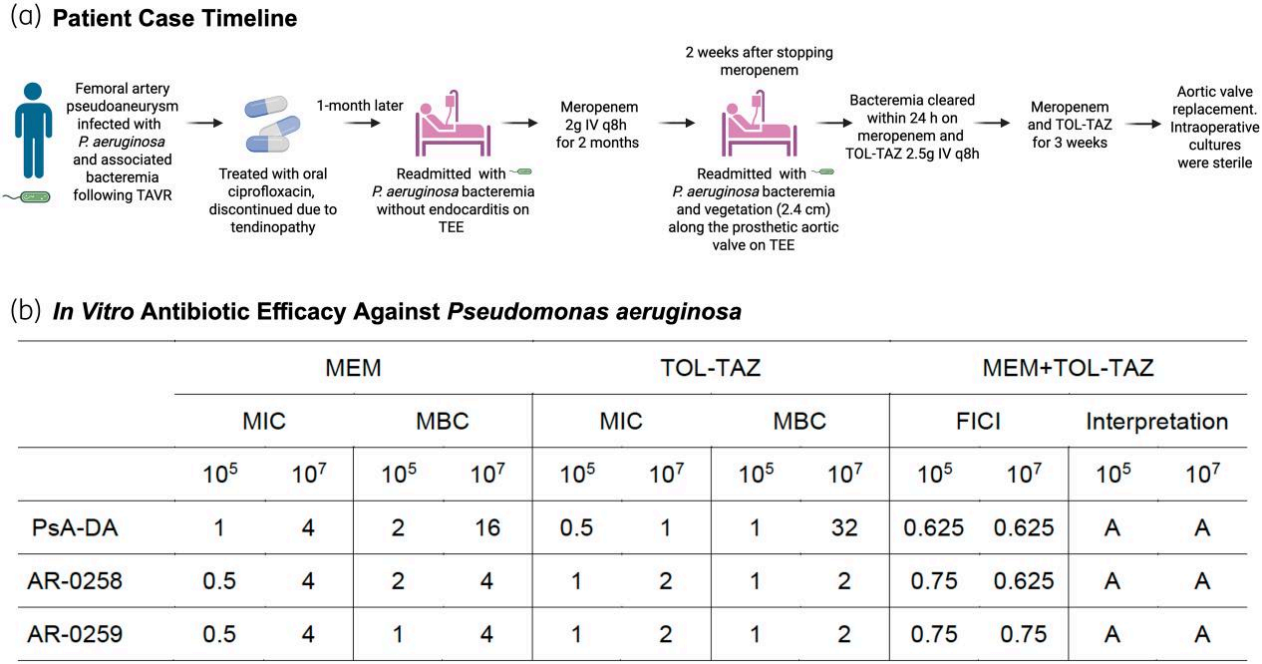
Clinical timeline and *in vitro* antibiotic efficacy of meropenem (MEM) and ceftolozane/tazobactam (TOL-TAZ) against *P. aeruginosa*. (a) Timeline of clinical events and antibiotic treatment for a patient with PVE caused by recurrent *P. aeruginosa* bacteraemia (PsA-DA) following TAVR. (b) MIC and checkerboard assays of *P. aeruginosa* isolates tested with MEM and TOL-TAZ under standard (10^5^ cfu/mL) and high (10^7^ cfu/mL) inoculum conditions. MICs and MBCs are expressed in mg/L. See Materials and methods for FICI interpretation criteria. Created in BioRender. Ulloa, C. (2025) https://BioRender.com/4xr1e71. TEE, transoesophageal echocardiogram; IV, intravenous; PO, per os (by mouth); FICI, fractional inhibitory concentration index; A, additivity.

A 77-year-old male with type 2 diabetes and hypertension underwent transcatheter aortic valve replacement (TAVR), complicated by a femoral artery pseudoaneurysm requiring graft repair. Blood and intraoperative cultures grew pan-susceptible *P. aeruginosa*, initially treated with ciprofloxacin (750 mg PO q12h), which was discontinued prematurely due to tendinopathy (Figure 1a). One month later, he again developed *P. aeruginosa* bacteraemia, requiring debridement and artegraft placement. Initial transoesophageal echocardiogram (TEE) was negative. He received 2 months of meropenem (2 g IV q8h), but bacteraemia recurred 2 weeks post-treatment. Combination therapy with meropenem (2 g IV q8h) and ceftolozane/tazobactam (2.5 g IV q8h, renally adjusted) cleared the bacteraemia within 24 h. A nuclear-tagged white blood cell scan showed no femoral uptake, whereas a subsequent TEE revealed a large (2.4 cm) prosthetic valve vegetation. Combination therapy was continued for 3 weeks as a bridge to aortic valve replacement, which yielded sterile cultures. Post-operatively, the patient received 3 weeks of ceftolozane/tazobactam followed by 5 weeks of meropenem, with no recurrence at 1-year follow-up. All clinical isolates had identical susceptibility profiles; the bloodstream isolate from his last admission (PsA-DA) was used for subsequent studies.

*In vitro* testing with PsA-DA and two comparator strains with similar susceptibilities showed an increase in the meropenem MIC from 0.5–1 to 4 mg/L under high inoculum (Figure 1b). Notably, PsA-DA also exhibited increased MBCs for meropenem and ceftolozane/tazobactam under these conditions. These inoculum effects highlight a critical limitation of standard MIC testing, which may fail to capture antibiotic tolerance in high-density *Pseudomonas* infections typical of endocarditis. This may explain the clinical reliance on high-dose and/or prolonged therapy in infections with substantial bacterial burdens.

**Figure 2.**
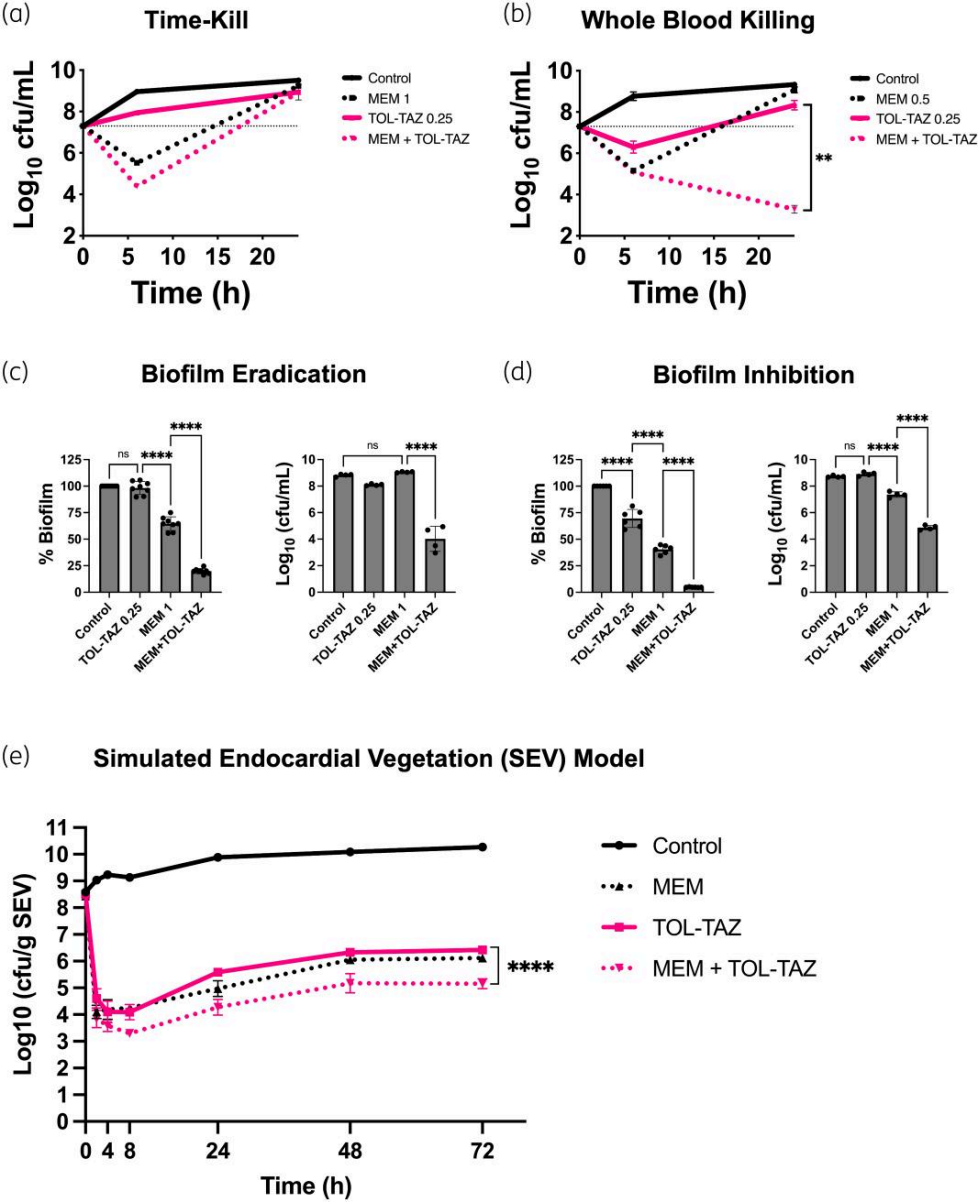
*In vitro* and *ex vivo* evaluation of meropenem (MEM) and ceftolozane/tazobactam (TOL-TAZ) against *P. aeruginosa*. (a) Time-kill assays of *P. aeruginosa* (PsA-DA) conducted at 6 and 24 h under high bacterial inoculum (2 × 10^7^ cfu/mL) in cation-adjusted Mueller–Hinton broth, using MEM (1 mg/L) and TOL-TAZ (0.25 mg/L) at 1/4 MIC. (b) Human whole blood killing assays assessing bacterial survival in the presence of MEM (0.5 mg/L, 1/8 MIC) and TOL-TAZ (0.25 mg/L, 1/4 MIC). (c) Assessment of bactericidal activity against preformed biofilms using MEM (1 mg/L) and TOL-TAZ (0.25 mg/L). (d) Evaluation of biofilm formation inhibition following antibiotic pre-treatment with MEM (1 mg/L) and TOL-TAZ (0.25 mg/L). (e) SEV model monitoring bacterial counts over 8–72 h. Statistical analysis: (a and b) **P ≤ 0.01, unpaired two-tailed *t*-test. (c and d) One-way ANOVA with multiple comparisons (****P ≤ 0.0001 or ns). (e) Two-way ANOVA with Tukey’s *post hoc* test (*P* = 0.0001).

To further probe therapeutic strategies under high-inoculum conditions, we next investigated the potential for synergy between meropenem and ceftolozane/tazobactam, as well as the impact of the host environment. Combination therapy revealed additive effects across all isolates and inocula in checkerboard assays (Figure 1b). Time-kill assays were performed to mimic worst-case scenarios, using high bacterial densities and sub-MIC antibiotic concentrations to model difficult-to-treat infections at poorly penetrated sites, aiming to reveal drug interactions masked at higher concentrations. While no synergy was observed in CA-MHB (Figure 2a), a 4 log_10_ cfu/mL reduction was achieved in whole blood at 24 h (Figure 2b). These results highlight the impact of host factors on antibiotic efficacy and suggest that standard *in vitro* assays may underestimate therapeutic potential under physiologic conditions.^6,11–14^

Biofilm assays also confirmed enhanced efficacy of combination therapy, with up to a 4-log₁₀ cfu/mL eradication (*P* ≤ 0.0001) and 2.5 log_10_ cfu/mL inhibition (*P* ≤ 0.0001) versus the most active monotherapy for PsA-DA (Figure 2c and d). Similar trends were seen for two other isolates (Figure S1). These synergistic effects are particularly relevant for IE, where biofilms promote antibiotic tolerance and immune evasion, often leading to treatment failures and high-risk surgery. Consistent with our findings, previous reports have demonstrated synergistic activity of meropenem and ceftolozane/tazobactam, including suppression of resistance in a hollow-fibre infection model of extensively drug-resistant strains.^15,16^

To assess translational efficacy at clinically relevant antibiotic exposures, we tested the combination in a simulated SEV model that replicates human pharmacokinetics and IE pathophysiology. In this model, combination therapy resulted in consistent 0.6–1.3 log_10_ cfu/g reductions from 8 to 72 h (*P* < 0.05; Figure 2e; Table S2). Despite early bacterial killing with all treatments, counts gradually rebounded by 24 h, yet remained 5 log_10_ cfu/g below controls. MICs remained unchanged, indicating that this increase may have resulted from antibiotic-tolerant persister cells or transient adaptive resistance. These findings suggest that optimizing dosing—through frequent or continuous infusions—may help sustain therapeutic levels and prevent recurrence.

While seemingly modest, the bacterial reductions observed with combination therapy may have significant clinical implications, given that culture detection limits typically range from 100 to 1000 cfu/g of tissue. In this context, a 90% cfu reduction could determine whether a valve culture is positive or negative at surgery. Valve culture results significantly impact patient outcomes, with positive cultures linked to increased mortality in surgical IE cases.^17,18^ Therefore, achieving a negative valve culture is critical and may also allow for shorter post-surgical antibiotic courses.^17^ Ultimately, strategies that promote negative valve cultures could improve patient outcomes, reduce adverse events and costs, and enhance antimicrobial stewardship.

In conclusion, our study provides compelling evidence supporting the efficacy of meropenem plus ceftolozane/tazobactam against biofilm-associated *P. aeruginosa* infections, particularly in IE. Although based on limited isolates, these results offer proof-of-concept for a potentially safer, more effective alternative to traditional regimens, and a possible bridge to valve replacement surgery. Given the paucity of large clinical trials addressing the management of patients with such severe infections, experimental laboratory analyses like ours will continue to play a critical role in guiding therapeutic decisions. Further studies are needed to confirm these findings across a broader range of *Pseudomonas* isolates, investigate the mechanisms underlying the observed synergy, and evaluate the clinical implications in patients with *P. aeruginosa* IE.

## Supplementary Material

dlaf112_Supplementary_Data

## Supplementary data

Figure S1 and Tables S1 and S2 are available as Supplementary data at *JAC-AMR* Online.
