# Supplementary material for: Rapid valve sterilization with meropenem plus ceftolozane/tazobactam combination therapy for Pseudomonas aeruginosa prosthetic valve endocarditis
Source: JAC Antimicrob Resist. 2025 Jun 26;7(3):dlaf112. doi: 10.1093/jacamr/dlaf112 (PMC12198497; doi:10.1093/jacamr/dlaf112)
Supplement: dlaf112_Supplementary_Data [file dlaf112_supplementary_data.pdf]

Supplemental Table 1. Minimum inhibitory concentrations (MIC, mg/L) and susceptibility interpretations for *Pseudomonas aeruginosa* clinical isolates.

**Antibiotic**	***Pseudomonas aeruginosa* PsA-DA**		***Pseudomonas aeruginosa* AR-0258***		***Pseudomonas aeruginosa* AR-0259***	
	MIC	Interpretation	MIC	Interpretation	MIC	Interpretation
Amikacin	≤16	Susceptible	≤2	Susceptible	≤2	Susceptible
Aztreonam	≤4	Susceptible	8	Susceptible	4	Susceptible
Cefepime	≤2	Susceptible	2	Susceptible	≤1	Susceptible
Ceftazidime	≤1	Susceptible	≤2	Susceptible	≤2	Susceptible
Ceftazidime/Avibactam	≤4	Susceptible	2	Susceptible	1	Susceptible
Ceftolozane/Tazobactam	≤2	Susceptible	≤0.5	Susceptible	≤0.5	Susceptible
Ciprofloxacin	≤0.25	Susceptible	≤0.25	Susceptible	≤0.25	Susceptible
Gentamicin	4	Susceptible†	---	---	---	---
Imipenem	2	Susceptible	2	Susceptible	2	Susceptible
Levofloxacin	≤0.5	Susceptible	0.5	Susceptible	0.5	Susceptible
Meropenem	≤1	Susceptible	≤0.25	Susceptible	0.5	Susceptible
Piperacillin/Tazobactam	≤8	Susceptible	≤4	Susceptible	≤4	Susceptible
Tobramycin	≤2	Susceptible†	≤0.5	Susceptible	≤0.5	Susceptible

*Adapted from the CDC and FDA Antibiotic Resistance Isolate Bank.

†In June 2023, the CLSI released updated aminoglycoside breakpoints for *Pseudomonas aeruginosa* as part of CLSI M100-Ed33. The MIC interpretations referenced were extracted from the electronic medical record prior to this update. Notably, gentamicin is no longer recommended as a treatment option for *P. aeruginosa*. Additionally, the susceptibility breakpoints for tobramycin have been revised: ≤1 mg/L is susceptible, 2 mg/L is intermediate, ≥4 mg/L is resistant.

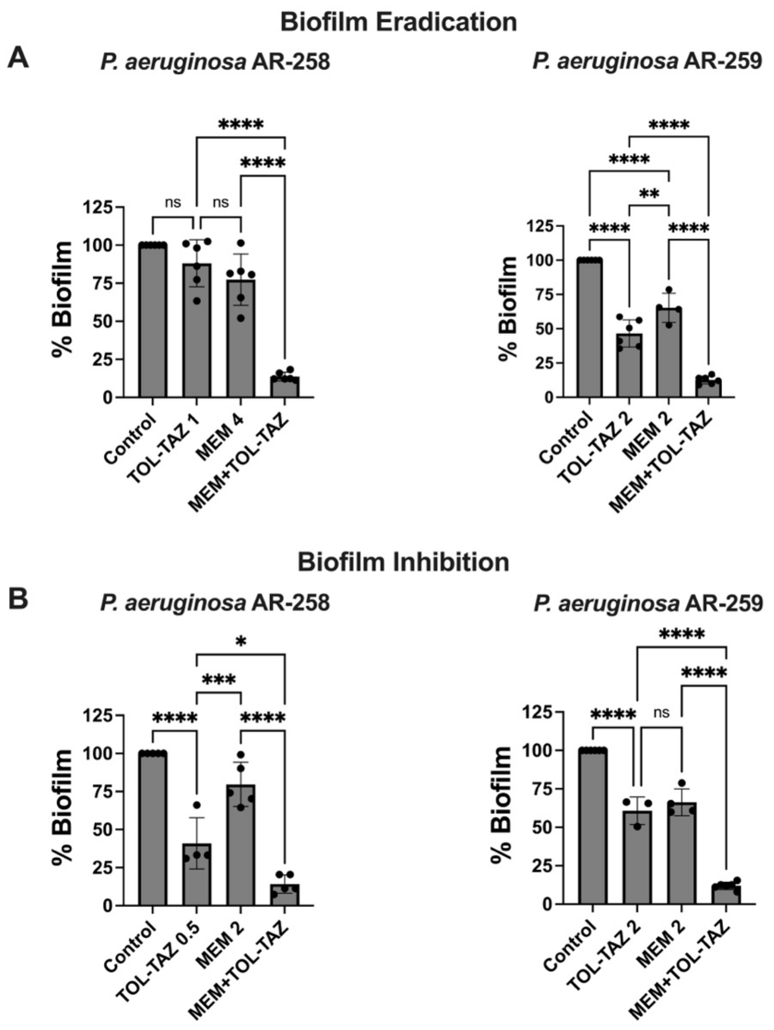

Supplemental Figure 1. Synergistic killing of *Pseudomonas aeruginosa* (AR-258 and AR-259) biofilms by meropenem (MEM) and ceftolozane/tazobactam (TOL-TAZ). **(A)** MEM and TOL-TAZ eradicated preformed biofilms, and **(B)** inhibited biofilm formation. Statistical analysis was performed using one-way ANOVA with multiple comparisons (****P ≤ 0.0001, ***P ≤ 0.001, **P ≤ 0.01, *P ≤ 0.05, or ns).

Supplemental Table 2. Bacterial quantification of *Pseudomonas aeruginosa* (PsA-DA) at each time point over 72 hours in the *ex vivo* simulated endocardial vegetation (SEV) model.

	**Time points (Log_{10} cfu/g, mean ± SD)**						
	**0h**	**2h**	**4h**	**8h**	**24h**	**48h**	**72h**
**Control**	8.58 ± 0.3	9.04 ± 0.32	9.24 ± 0.04	9.14 ± 0.03	9.88 ± 0.11	10.09 ± 0.25	10.27 ± 0.07
**MEM**	8.44 ± 0.04	4.11 ± 0.49[a]	4.19 ± 0.73[a]	4.25 ± 0.14[a]	4.97 ± 0.60[a]	6.05 ± 0.08 [a]	6.11 ± 0.08[a]
**TOL-TAZ**	8.51 ± 0.03	4.61 ± 0.72[a]	4.10 ± 0.81[a]	4.10 ± 0.58[a]	5.60 ± 0.13[a]	6.33 ± 0.19 [a]	6.42 ± 0.11[a]
**MEM+TOL-TAZ**	8.51 ± 0.01	3.84 ± 0.64[a]	3.59 ± 0.45[a]	3.30 ± 0.30[a,b,c]	4.28 ± 0.60[a,b]	5.17 ± 0.72 [a,b,c]	5.16 ± 0.37[a,b,c]

The mean log_{10} cfu/g ± SD is presented for each time point (n = 4). Statistical significance is indicated as follows: [a]$P < 0.05$ vs control; [b]$P < 0.05$ vs TOL-TAZ; [c]$P < 0.05$ vs MEM. A two-way ANOVA with Tukey's post-hoc test was used to compare bacterial counts at each time point.

Abbreviations: MEM, meropenem; TOL-TAZ, ceftolozane/tazobactam.

Notes: In this simulation, antibiotic concentrations were adjusted to match target drug estimates, considering protein binding in the SEV model.[9-13] For meropenem, the Cmax was 98 µg/mL, half-life ($t_{1/2}$) was 1 hour, and Cmin was 0.4 µg/mL.[9] For ceftolozane, the Cmax was 105 µg/mL, $t_{1/2}$ was 3.7 hours, and Cmin was 21 µg/mL.[10,11] Tazobactam was excluded from the simulation due to limited activity against *P. aeruginosa*.[10,12] For the meropenem plus ceftolozane/tazobactam combination, we used a modeling approach designed for antibiotics with different half-lives.[13] Antibiotic regimens were simulated over 72 hours, with SEV samples taken at 2, 4, 8, 24, 48, and 72 hours.
